# The awareness of renal stones amongst Syrian refugees in northern Jordan

**DOI:** 10.1371/journal.pone.0300999

**Published:** 2024-06-14

**Authors:** Hakam Alaqabani, Hani Omar, Sara Yaser Barham, Hashim H. Al Zuaini, Agata Ugorenko, Anas Khaleel

**Affiliations:** 1 Faculty of Pharmacy, Al-Zaytoonah University of Jordan, Amman, Jordan; 2 Department of Pharmacy and Biomedical Sciences, University of Strathclyde, Glasgow, United Kingdom; 3 Faculty of Information Technology, Zarqa University, Zarqa, Jordan; 4 Department of Molecular Medicine, Faculty of Medicine, Yeditepe University, Istanbul, Turkey; 5 Department of Pharmacology and Toxicology, Faculty of Pharmacy, Mustansiriyah University, Baghdad, Iraq; 6 Department of Pharmacology and Biomedical Sciences, Faculty of Pharmacy and Medical Sciences, University of Petra, Amman, Jordan; Jordan University of Science and Technology, JORDAN

## Abstract

Kidney Stone Disease (KSD) is a globally prevalent condition that can be effectively addressed through proper education. This study investigated the awareness of kidney stones among refugees residing in northern Jordan. A questionnaire was administered to 487 refugees of diverse ages and socioeconomic backgrounds. Notably, 97.3% of the respondents had not attended university, and 30.8% held unskilled jobs. Upon familiarizing themselves with the signs and symptoms of kidney stones, 16.22% of men and 12.32% of women reported experiencing such symptoms. This revealed a significant association, suggesting that men might be more susceptible to developing kidney stones than women due to a lack of medical follow-up and examination for men in the camp. However, 38.77% of individuals were uncertain whether they had kidney stones. Furthermore, 38.96% of refugees were unsure about which healthcare professional to consult when experiencing kidney stone symptoms. This report highlights a serious issue with refugees’ knowledge of the symptoms, causes, and treatments for kidney stones. The results indicate that Syrian refugees face challenges in acquiring adequate disease awareness, potentially related to issues of migration and war, including low levels of education, limited income, living in camps, and difficulties accessing treatments when needed. Implementing additional policies is necessary to address these challenges among Syrian refugees; however, further studies are needed to validate these findings.

## Introduction

Health systems and humanitarian organizations that help refugees in Jordan face many challenges—providing cost-efficient, integrated methods to achieve stability in the management of diseases, ensuring that medication is consistently administered and accessible, providing patient education, and managing acute conditions such as diabetic complications which include cardiac disorders, renal insufficiency, neuropathy, and additional conditions [1]. The current political climate sees an influx of refugees fleeing their countries and being placed in temporary or permanent camps in different countries. The Zaatari refugee camp is the biggest camp for Syrian refugees, located 10 kilometers east of Irbid, the northern Jordanian city, and it hosts over 82000 refugees, leading to the refugees sharing the same social, psychological, environmental, and health care conditions [2]. The prolonged displacement could result in the development of non-communicable diseases, which, paired with a lack of diagnosis and awareness of their condition, in addition to the shortage of data about camps, could result in an increased disease burden for the refugees, with potentially severe outcomes caused by untreated diseases [3].

Kidney stones are among the diseases observed in refugee communities. Many refugees suffer from kidney stone symptoms, as well as a lack of understanding about the causes, symptoms, and management. Furthermore, camp clinics lack the necessary equipment to diagnose kidney stones, and clinics are satisfied with treating symptoms and dealing with critical disease cases after the symptoms worsen, as the patients’ suffering continues, affecting their quality of life and increasing the cost for organizations to treat disease exacerbations [4].

Nephrolithiasis refers to renal stones that form in the urinary tract when a mineral in the urine becomes oversaturated, causing crystal formation, aggregation, and retention within the kidneys, which can lead to the development of kidney stone disease (KSD). It affects people of all ages and genders [5, 6]. The formation of urinary stones (urolithiasis) is a complex process, and it can cause discomfort and painful recurrence, although people can develop and pass these stones while still asymptomatic [7]. Often, these stones require costly surgical removal, and in recent years, kidney stones have become more common in the general population around the world [8]. The prevalence of kidney stones differs significantly worldwide, with Asia reporting 1–5%, North America at 7–15%, and Europe at 5–9% of the population suffering [9]. However, a study published in the Jordan Medical Journal in 2017 reported that the prevalence of kidney stones in Jordan was 7.6% [10].

Furthermore, kidney stones are prone to return, with a projected recurrence rate of 50% following ten years of treatment [11]. The research undertaken and the wide variation in rates and incidences of kidney stones reported suggest an increase in the cases in recent years, which leads to the strain of providing healthcare worldwide. The cost varies from person to person and from technique to technique in terms of diagnosis, examinations, appropriate treatment, and, if necessary, surgery [12]. KSD is also linked to an elevated risk for end-stage renal disease (ESRD) [13] and chronic morbidity, including a reduction in bone density, cardiovascular disease, and chronic kidney disease (CKD) which is caused by uric acid stones [14, 15] The disease itself, as well as comorbidities, have a negative effect on healthcare, as well as cost additional money for more advanced treatment which can especially affect vulnerable communities with little-to-no access to healthcare providers or hospitals. Therefore, it is crucial to educate the public on the prevention of kidney stones and the early detection of symptoms [1]. A variety of reasons can cause kidney and urinary stones. It has been reported that modifiable factors, particularly environmental and dietary ones, are associated with the likelihood of developing kidney stones, including being overweight or obese, the quantity and composition of fluids consumed, the DASH diet a dietary regimen abundant in low-fat dairy products, fruits, and vegetables and the amount of dietary calcium consumed [16] influenced by metabolic, genetic, nutritional, anatomical structure [17, 18] as well as family history [19], manual labor employment [20], and socioeconomic profiles [21].

The formation of kidney stones may also be influenced by gender; it is reported that approximately 5% of women and 12% of men will develop kidney stones during their lifetime. This is due to several factors, such as hormonal factors, as the level of testosterone is higher in men, and that can increase the levels of calcium in the urine and make it more likely for stones to form [22]. Non-communicable disease (NCD) management has not been a priority in humanitarian settings, contributing to the spread of diseases such as kidney stones. A comprehensive understanding of factors influencing kidney stone formation is essential. This research focuses on examining the awareness of kidney stone disease among Syrian refugees in Northern Jordan, aiming to characterize knowledge and awareness in the Zaatari camp where information on kidney stone illness in Syrian refugees is lacking.

## Methods

### Study design

The present report used a cross-sectional approach. A brief survey was used to collect information about kidney stones [23]. Ethical and related approvals were taken to conduct this research from The Directorate of Syrian Refugee Affairs—the Jordanian Ministry of Interior, the Research Ethics Committee, the Directorate of Medical Education, Training, the Directorate of Project Management, Planning and International Cooperation, MOH/REC/2022/162 and Al-Zaytoonah University also approved this research. In addition, written consent was obtained from the questionnaire participants. The survey samples were obtained by randomly selecting a number of refugees from the Zaatari refugee camp based on the population distribution of refugees in the camps’ sectors and by selecting one person over 18 per house for the in-person questionnaire.

A validated survey, which focused on the incidence of kidney stone disease and public knowledge about the condition, was conducted. The survey was translated into Arabic, the native language of Syrian refugees, and it was filled out by individuals in the general population. According to The United Nations High Commissioner for Refugees (UNHCR), in January 2023, the camp was home to almost 82,679 Syrian refugees (almost 19,500 families) with 32 schools, eight health facilities, and 58 community centers. Samples were taken based on the distribution of the population inside the camp in sectors from 1 to 10, with an average of 50 people in each sector with a total number of 487. Data was collected from the Syrian refugee population from September through December 2022.

### Statistical analysis

A survey was established and revised from the literature. The analysis of all data was performed using IBM® SPSS® software, version 27, based in Chicago, IL, USA.

### Sample size

Slovin’s formula and Krejcie and Margon’s formula were used to determine sample size. The population size was estimated at 81,000 refugees [24], the confidence interval (CI) was 0.95, and the margin of error was 5%. A sample size of 383 subjects was required to achieve the required CI. In this study, 487 participants were enrolled. There were no prerequisites for refugees to participate in the study other than the fact that they had to be older than 18, and those who refused to respond to the questions were disqualified.

## Results and discussion

A total of 487 respondents (299 males and 186 females) completed the survey, providing information on their demographic and lifestyle factors. The survey included questions about age, gender, marital status, weight, smoking habits, and reported diseases, as detailed in Table 1. The participants were primarily middle-aged (average 39 years old ±16.50), overweight (average BMI 28.63±3.97), and non-smoker (78%).

**Table 1:** Participant demographics.

Demographic Characteristics			Sample n (%)
**Age** $\bar{x}$ **± SD (years)**			39.44 (±16.50)
**Sex**	**Male**		299 (61.4%)
	**Female**		188 (38.6%)
**Marital status**	**Married**		281 (57.7%)
	**Not married**		206 (42.3%)
**Body mass index**		**Male**	**Female**
	**Underweight**	1.06%	0.49%
	**Normal weight**	51.06%	46.50%
	**Overweight**	39.90%	39.31%
	**Obese**	7.98%	13.70%
**Smoking status**	Nonsmoker		78%
	**Smoker**		22%
**Type of smoking for smokers**	**Cigarette**		71.96%
	**Hubbly bubbly**		2.80%
	**Electronic cigarettes**		25.24%

n, number of samples; $\bar{x}$ ± SD, mean value ± standard deviation.

The percentage of obese females was 13.7%, which is more than that of males, while the majority of men and women had normal or overweight weight. The higher rates of obesity in women compared to men are influenced by several factors, among them biological, cultural, and societal factors. The hormonal fluctuations linked to the menstrual cycle, pregnancy, and menopause can impact a woman’s metabolism and energy balance [25].

Higher body weight may result in a rise in the amount of calcium expelled through urine, which can, in turn, increase the likelihood of developing kidney stones. Additionally, the increased insulin resistance that often accompanies obesity can modify urinary chemistry, potentially elevating the chance of developing kidney stones [26].

The Information on the educational background and occupation of the participants was gathered in Table 2.

**Table 2:** The distribution of jobs and educational levels among Syrian refugees.

A) Occupation	A) Sample %	B) Education level	B) Sample %
**Skilled workers**	14.40%	**Elementary or less than high school**	86.94%
**Nonskilled worker**	30.80%		
		**High school**	10.35%
**Student**	5.70%		
**Housewife**	34.70%	**Higher than high school (Diploma, Bachelor, Master or PhD)**	2.71%
**Non-worker**	14.40%		

A) Syrian refugees in the Zaatari camp work non-skilled jobs 30.8%, with only 13.1% working skilled jobs or pursuing further education. A total of 34.7% declared to work as a housewife, with 14.4% non-workers. B) Most of the Syrian refugees in the Zaatari camp had high school or lower education; this is in line with the workforce distribution reported by the participants, as housewives and jobs requiring no skills/experience were amongst the most common in the camp. The majority 97.3% of participants declared to have education at the high school level or lower, with only 2.71% having higher education (diploma, bachelor, master or PhD).

The research shows that lower education leads to lower occupation, which contributes to lower income and poverty; it is also an indicator of low socioeconomic status (SES), which can lead to the development of non-communicable diseases such as kidney stones. [18, 19]. Both in low- and middle-income countries, as well as developed nations, patients with lower socioeconomic status (SES) suffer from a lot of diseases such as kidney disease (kidney stones, urinary stones), cardiovascular diseases (hypertension), obesity, and diabetes [20, 21]. Individuals with limited educational backgrounds may have reduced access to resources and knowledge regarding healthy dietary habits, which can increase the likelihood of developing kidney stones. For example, a diet rich in salt, animal protein, and sugar can be a contributing factor to kidney stone formation, and individuals with limited education may have insufficient awareness of these dietary risks. Moreover, they may encounter barriers in accessing healthcare services, which can impede the diagnosis and treatment of kidney stones. This can result in delayed treatment and potentially severe complications [27].

**Fig 1:**
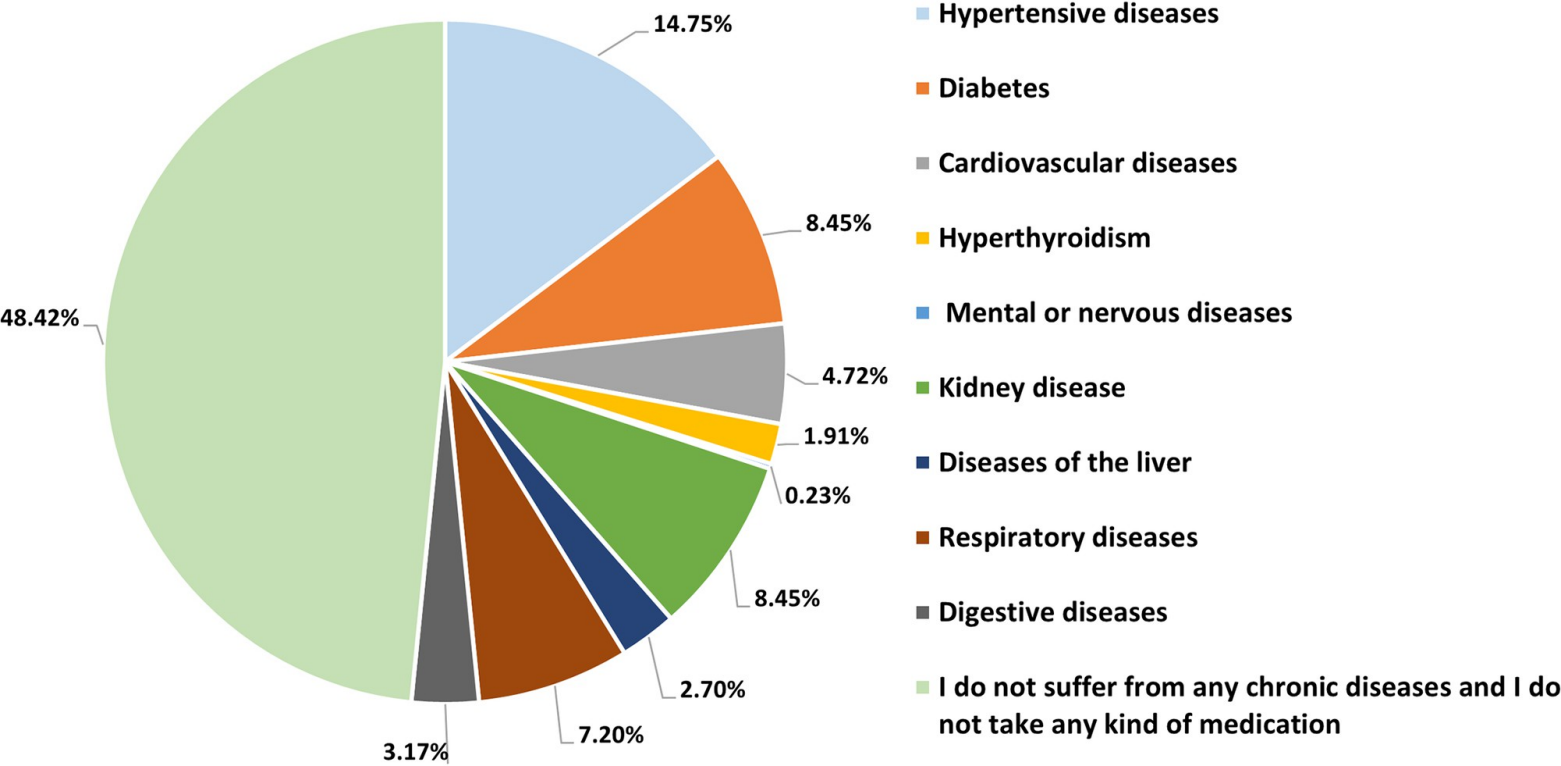
Prevalence of chronic diseases and conditions among Syrian refugees.

Participants were prompted to disclose any health conditions or chronic diseases they may be experiencing, as indicated in Fig 1.

Hypertensive diseases, diabetes, and kidney diseases were among the three most prevalent conditions among participants, constituting 31.65% of responses. Other health conditions such as respiratory diseases, cardiovascular diseases, liver diseases, mental/nervous diseases, hyperthyroidism, and digestive issues plagued 19.93% of participants, whereas 48.42% of participants did not suffer from the mentioned diseases.

Our findings show that a large percentage of participants suffer from chronic health conditions or diseases. It is important to note that the above percentages refer to those diagnosed with these diseases. Still, there is a possibility that a large number of refugees have these diseases but have not been diagnosed. This could be caused by some of the factors mentioned in previous studies, including the refugees’ fear of being diagnosed, their reluctance to take medications because of their side effects, the lack of access to some medications and medical care, costs associated with treatment, and the patient’s potential waiting period of several months, which leads to a shortage in diagnosing diseases among some refugees [28].

These findings and percentages are comparable with earlier research by A.M. HAMMAD (2022), which found that hypertension and diabetes are the most prevalent diseases among Syrian refugees and have proportions that are somewhat close to those in this study [29].

There is some evidence to suggest that having a history of kidney stones in the family could increase the likelihood of developing kidney stones, which may be due to inherited factors, like genetic predisposition to certain stone-forming substances or to metabolic abnormalities that increase the development of kidney stones [30]; therefore the participants were asked if they or their family members currently have or suffered in the past from kidney stones. A total of 487 participants were interviewed, and the results, as shown in Table 3, revealed that 8.45% of them reported a diagnosed history of kidney stones. In contrast, 21.76% reported a family member with a history of the disease. On the other hand, 12.12% of the participants reported never having had kidney stones, while 18.90% reported no family member had a history of the disease. Moreover, 38.77% of the participants were uncertain whether they had kidney stones, possibly because the symptoms can be similar to other conditions, such as a urinary tract infection or muscle strain [31]. This uncertainty could be attributed to a lack of education and knowledge about the disease, as reflected in the data presented in Table 3. Additionally, it is worth noting that some individuals may have small kidney stones that pass through the urine without causing noticeable symptoms [32].

**Table 3:** Gender-related association with kidney stone diagnosis and associated symptoms.

Diagnosed with kidney stones (8.45%)		No diagnosis with kidney stones (91.55%)			
Male	23.07%	**Disease symptoms are present**		**No symptoms of the disease**	
Female	76.93%	Male	16.22%	Male	38.19%
		Female	12.32%	Female	33.27%

There is a difference in the proportion of women diagnosed with kidney stones in comparison to men; however, more men than women suffered from kidney stone symptoms without a diagnosis. More men had not experienced kidney stone symptoms in the unaffected population than women. Contrary to what was predicted based on prior studies, which revealed a greater incidence of kidney stones in males [25], in this study, more females than males were suffering from kidney stones. However, men were more likely to suffer kidney stone symptoms without a diagnosis, suggesting that women received better diagnoses than men. As a result, the proportion of men who experience kidney stone symptoms may be higher or equal to that of women.

Certain foods can contribute to the development of kidney stones, especially if consumed in excess, such as foods high in oxalate, which include spinach, rhubarb, beets, nuts, and chocolate. When oxalate levels become too high, it can bind with calcium to form crystals that can lead to the formation of kidney stones [33]. Other foods that can lead to kidney stone formation are those high in purines, such as organ meats, anchovies, sardines, herring, and mussels. Purines can be metabolized into uric acid in the body, and an excess amount of them in the urine leads to the development of uric acid stones [34]. A diet high in sodium can also increase the amount of calcium in the urine, which can contribute to the formation of calcium stones [35].

**Fig 2:**
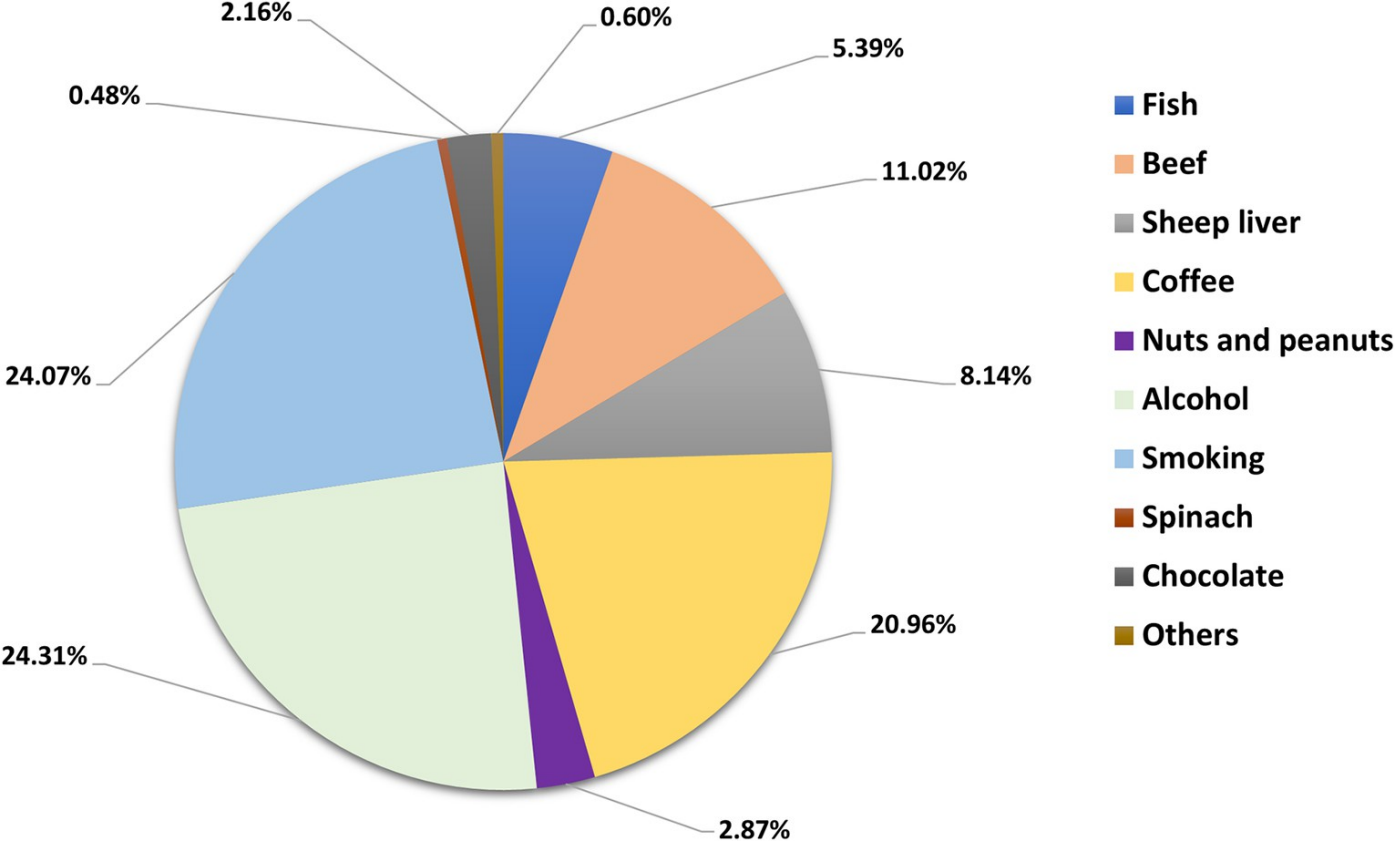
Participant perspectives on nutritional factors contributing to kidney stones.

The participants were asked about the consumption of food, smoking, and alcohol to gauge their understanding of potential causes of the development of kidney diseases see Fig 2. A total of 24.31% of participants believe alcohol to be the cause of kidney stones, followed by smoking 24.07% and coffee consumption 20.96%. Meat and fish consumption was named as a cause by 24.55% of participants, whereas nuts and peanuts, spinach, and chocolate were only named by 5.51% of responders.

Participants identified alcohol as a primary factor contributing to the formation of kidney stones. The study indicates that a heightened risk is associated with heavy alcohol consumption, particularly exceeding 3 to 4 alcoholic drinks per day [36]. A total of 24.07% of participants chose smoking as a potential cause of kidney stones. Based on previous studies, smoking increases the incidence of kidney stones; refugees opted for this because they typically hold the view that smoking is harmful to their health. Coffee consumption placed third as the potential cause of kidney stone formation. A study by Shuai Yuan (2022) [37] showed that coffee and caffeine lower the risk of kidney stones because coffee can increase urine production and is a mild diuretic, meaning it can increase urine output. This can aid in preventing the buildup of minerals and crystals in the kidneys, which can lead to the formation of stones [37]. This suggests that Syrian refugees are unaware of the link between coffee and kidney stones. Participants also chose beef (11.02%), sheep liver (8.14%), and fish (5.39%) as potential causes of the stones forming. These products increase the incidence of kidney stones, as shown by previous studies. Interestingly, only a small fraction of participants chose chocolate (2.16%).

These results indicate a lack of awareness of the effect of products consumed on the formation of kidney stones among Syrian refugees. Alcohol and smoking, which were leading causes picked by participants, are universally seen as unhealthy, which could potentially lead to them being picked rather than actual knowledge. Therefore, education must be easily accessible in the hope of avoiding the development of kidney stones.

Participants have also shared their smoking habits see Table 1. Refugees believed smoking to be one of the leading causes of kidney stone formation see Fig 2, and this translates to the percentage of smokers in the group, as the majority, 78% of participants, are nonsmokers. Cigarettes are most popular amongst smokers (71.96%), followed by electronic cigarettes (25.24%) and hubby bubbly (a smoking device that consists of single or multiple stems used for heating or vaporizing and then smoking tobacco or flavored tobacco) 2.8%. However, the low percentage of smokers could be caused by reasons not associated with health—the difficult financial situation and the high costs associated with smoking could be another reason [38].

**Fig 3:**
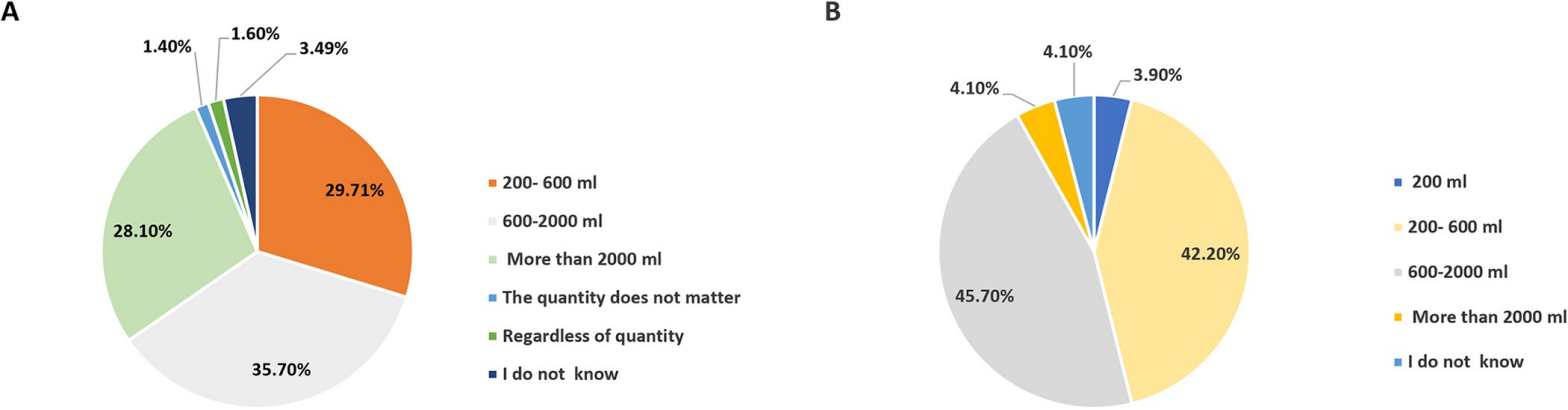
Perspectives of refugees on recommended daily water intake and their actual water consumption. (A) Participant beliefs regarding daily fluid intake to prevent kidney stone formation (B) Participant reported water consumption levels.

Drinking plenty of water is one of the most effective ways to prevent kidney stone formation; it helps dilute the concentration of substances in the urine that can form stones, making it less likely that stones will form. In addition, drinking water helps increase urine volume, which can help flush out any substances that might be forming into stones [39]. The participants were asked about their perceptions of how much fluid intake is needed to avoid the formation of kidney stones see Fig 3.

A) A total of 63.8% of participants believed that more than 600mL of fluids a day is needed to stop the formation of kidney stones, 29.71% believe 200-600mL to be enough, while 35.7% believed 600-2000mL to be the appropriate amount. 28.1% regarded 2000mL as the minimum volume of liquids needed to stop the formation of stones. B) The majority of the participants were found to consume 600-2000mL of liquids a day (45.7%), with only 4.1% consuming above 2000mL. 42.2% drank 200-600mL liquids daily, with as much as 3.9% consuming 200mL or less. 4.1% did not know their liquid consumption.

There seems to be an association between the belief in the appropriate intake of fluids in a day and the actual consumption by the participants. The majority of the participants believed that 600-2000mL is enough to prevent the formation of kidney stones, and that’s the amount drank by most. However, based on previous clinical studies, the number of fluids the body requires to prevent kidney stones from forming was more than 2000 mL [40], which is the amount that 4.1% of refugees chose fluid intake and 28.1% chose this amount to prevent the formation of kidney stones.

These results further show participants’ lack of information and knowledge on preventing kidney stone formation.

We also asked the participants if calcium consumption is associated with the development of kidney stones and if holding urine can cause them.

The study showed that 80.7% of participants did not choose calcium as the cause of kidney stone formation, while only 19.3% thought it could cause kidney stones. The majority of participants believing that calcium does not cause kidney stones is in opposition to the results of previous studies, as calcium is a very important factor in the formation of kidney stones. Usually, excess calcium is excreted in the urine, but when there is too much calcium, it can combine with other substances to form crystals that can grow into stones. The type of kidney stone that occurs most frequently is the one made of calcium oxalate [40].

The majority of the refugees (65.5%) believe that there is no relationship between holding urine in the bladder and the formation of kidney stones. However, based on previous studies, holding urine may lead to the development of kidney stones. When urine is held in the bladder for long periods, it can become more concentrated, which means that the minerals and other substances in the urine also become more concentrated. This can increase the likelihood that these substances will form crystals, which can grow into stones [41].

**Fig 4:**
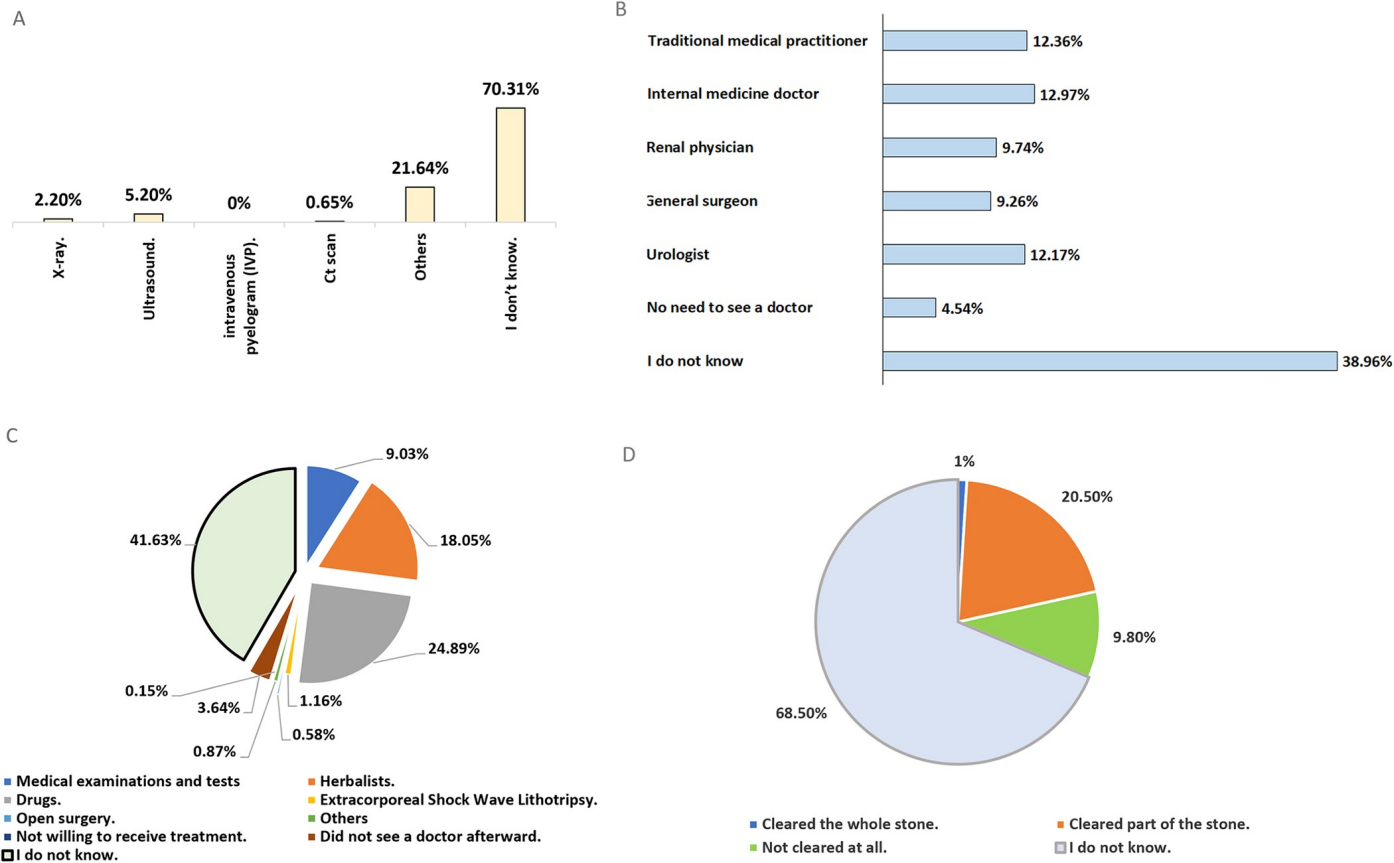
Participant awareness of kidney stone diagnosis, treatment, healthcare providers, and persistence. (A) the diagnosis of kidney stones. (B) healthcare providers involved in diagnosing kidney stones. (C) methods of treating kidney stones. (D) the determination of whether kidney stones have been successfully cleared or not.

As early diagnosis is important in treating kidney stones, we asked the participants which doctor can make the diagnosis and what the diagnostic process is; we also asked about the medications and treatments they are aware of see Fig 4.

Refugees’ opinions about a) diagnosis of kidney stones, b) doctors and processes used to diagnose kidney diseases, c) kidney stones treatment, and d) clearance of kidney stones.

The diagnosis of kidney stones typically involves a combination of medical history, with the doctor asking about the symptoms and family history, physical examination, and imaging tests [42]. The urine tests may be done to check for blood, infection, or crystals that may indicate the presence of a kidney stone. Finally, imaging tests are used to visualize the kidneys and urinary tract to confirm the presence of a kidney stone. The most common imaging tests used to diagnose kidney stones are X-ray, Ultrasound, and CT scan [43, 44], but according to our study, 70.31% of the participants didn’t know how kidney stones were diagnosed.

The results revealed a significant association between refugees’ knowledge of the appropriate healthcare provider for symptoms of illness and the diagnostic methods applied for kidney stones, along with educational attainment, resulting in a p-value of 0.056. This suggests that 38.96% of participants lacked awareness regarding both the physicians and procedures involved in diagnosing kidney diseases. This observation may be attributed to the participants’ educational attainment, as indicated in Table 2, where a significant proportion holds a high school diploma or lower. The underlying factors include challenging living conditions experienced by refugees, including migration and wars, which impeded their ability to complete their studies. Furthermore, limited financial resources contribute to this educational pattern [43].

The majority of the participants (41.63%) were unaware of the medications available for the treatment of kidney stones with as many as 18.05% using medicinal herbs for treatments. Moreover, 9.03% believed that monitoring the disease with periodic medical examinations and tests is enough, and no other types of treatments are needed. Interestingly, in terms of treatments, only 1.16% of participants chose extracorporeal shock wave lithotripsy (ESWL) employing laparoscopy, while open surgical received was chosen by 0.87% of participants, even though these are one of the best treatment options for kidney stones. Nevertheless, treatment options are constrained given the distinct characteristics of the camp, including financial constraints, insufficient clinic facilities, a high patient volume, and the prevalence of primary centers and hospitals. Consequently, surgical services for addressing kidney stones are often unavailable in these camps. Additionally, only 1% of refugees reported the removal of kidney stones after undergoing treatment. Furthermore, 68.5% of refugees were uncertain about the status of their stones post-treatment—whether they had been removed or were still present in their bodies. The persistence of kidney stones can lead to diverse symptoms and complications, such as pain and potential recurrence in the future. This, in turn, may impose additional financial burdens on individuals and healthcare systems [5]. This further demonstrates the lack of knowledge among the refugees on their level of sickness recovery. As shown before, this could be due to low education levels among the participants.

The lack of health education and awareness among refugees is a major issue despite the presence of health cadres for treatment. This can result in refugees experiencing common health problems such as kidney stones, which can be avoided through proper health practices. Therefore, this paper provides practical recommendations to improve the health and well-being of refugees, including seeking reliable sources of health information, maintaining a healthy diet, staying hydrated, practicing good hygiene, seeking medical help when needed, staying active, and building social connections, in addition to holding awareness lectures on the problem of kidney stones by non-profit organizations. Organizations dedicated to raising awareness conduct educational sessions on chronic diseases or birth control, but their scope is constrained when it comes to topics explicitly related to kidney health.

By prioritizing their health and well-being, refugees can reduce their risk of common health problems and improve their overall quality of life. However, it is not enough to solely provide individual-level health education. Organizational efforts are needed to improve the health system for refugees, as this can benefit refugees and the organizations that support them. By promoting greater health awareness and education among refugees, organizations can reduce the suffering and improve the lives of those they serve. In addition, this can also help to reduce the high cost of treatment that can result from preventable health problems.

Overall, the importance of lifelong health education for refugees cannot be overstated. By staying informed and making better decisions about their health, refugees can lead healthier and more fulfilling lives. Organizations that support refugees must prioritize these efforts, as they can significantly impact the health and well-being of this vulnerable population.

## Conclusion

In summary, this report suggests that Syrian refugees may lack adequate knowledge about the symptoms, causes, and treatments for kidney stones, which may be attributed to their challenging living conditions, low levels of education, lack of income, and limited access to healthcare services. The findings underscore the need for policies aimed at improving disease awareness among Syrian refugees, particularly in terms of enhancing their education, increasing their exposure to medical professionals, and promoting early diagnosis and treatment of kidney stones. However, further research is required to validate these results and explore additional measures that can help alleviate the difficulties faced by this vulnerable population.

## Supporting information

S1 Data (XLSX)
